# *BECN1* and *BRCA1* Deficiency Sensitizes Ovarian Cancer to Platinum Therapy and Confers Better Prognosis

**DOI:** 10.3390/biomedicines9020207

**Published:** 2021-02-18

**Authors:** Amreen Salwa, Alessandra Ferraresi, Menaka Chinthakindi, Letizia Vallino, Chiara Vidoni, Danny N. Dhanasekaran, Ciro Isidoro

**Affiliations:** 1 Laboratory of Molecular Pathology, Department of Health Sciences, Università del Piemonte Orientale “A. Avogadro”, Via Solaroli 17, 28100 Novara, Italy; amreensalwa12@gmail.com (A.S.); alessandra.ferraresi@med.uniupo.it (A.F.); meni23ram@gmail.com (M.C.); letizia.vallino@uniupo.it (L.V.); chiara.vidoni@med.uniupo.it (C.V.); 2 Stephenson Cancer Center, The University of Oklahoma Health Sciences Center, Oklahoma City, OK 73104, USA; Danny-Dhanasekaran@ouhsc.edu

**Keywords:** personalized medicine, ovarian cancer, chemoresistance, genome sequencing, tumor suppressor genes, autophagy, apoptosis, prognosis, epigenetics

## Abstract

Background: *BRCA1, BECN1* and *TP53* are three tumor suppressor genes located on chromosome 17 and frequently found deleted, silenced, or mutated in many cancers. These genes are involved in autophagy, apoptosis, and drug resistance in ovarian cancer. Haploinsufficiency or loss-of-function of either *TP53*, *BRCA1* or *BECN1* correlates with enhanced predisposition to cancer development and progression, and chemoresistance. Expectedly, the combined altered expression of these three tumor suppressor genes worsens the prognosis of ovarian cancer patients. However, whether such a genotypic pattern indeed affects the chemo-responsiveness to standard chemotherapy thus worsening patients’ survival has not been validated in a large cohort of ovarian cancer patients. Aim: We interrogated datasets from the TCGA database to analyze how the expression of these three tumor suppressor genes impacts on the clinical response to platinum-based chemotherapy thus affecting the survival of ovarian cancer patients. Results and conclusion: Compared to EOC with homozygous expression of *BECN1* and *BRCA1*, tumors expressing low mRNA expression of these two tumor suppressor genes (either because of shallow (monoallelic) co-deletion or of promoter hypermethylation), showed higher sensitivity to platinum-based therapies and were associated with a better prognosis of ovarian cancer-bearing patients. This outcome was independent of *TP53* status, though it was statistically more significant in the cohort of patients with mutated *TP53*. Thus, sensitivity to platinum therapy (and probably to other chemotherapeutics) correlates with low expression of a combination of critical tumor suppressor genes. Our study highlights the importance of thoroughly assessing the genetic lesions of the most frequently mutated genes to stratify the patients in view of a personalized therapy. More importantly, the present findings suggest that targeting the function of both *BECN1* and *BRCA1* could be a strategy to restore chemosensitivity in refractory tumors.

## 1. Introduction

Ovarian cancer ranks as the eighth leading cause of cancer-related deaths among women, and a leading cause of death from gynecological cancer [1]. The majority (up to 90%) of ovarian cancers are epithelial ovarian cancers (EOC) [2]. Ovarian cancer remains one of the most lethal gynecologic malignancies with a 5 years of survival rate lower than 50%, due to the lack of effective diagnosis of the disease at early stage and the persistence of drug-resistance [3,4]. Currently, the standard of care for EOC patients includes primary cytoreductive surgery followed by a platinum-based chemotherapy, that is considered as first-line treatment. Drugs such as cisplatin, oxaliplatin and carboplatin exert an anti-tumor effect by inducing intra-strand and inter-strand cross-links in genomic DNA and are cell cycle-non-specific in action. Although many patients initially respond to the treatment, more than 75% of high grade serous ovarian cancer (HGSOC) patients develop drug resistance within one year and eventually relapse [5,6]. Understanding the gene signature underlying chemoresistance would help to design personalized therapeutic interventions in ovarian cancer patients.

Based on the genetic mutations and clinical features, EOC are classified in Type-I and Type-II [7]. Type-I tumors are indolent, low progressing with relative genomic stability. These tumors frequently present mutations in *KRAS, BRAF, PTEN, PI3KCA* and *ERBB2* genes [7]. In contrast, Type-II tumors have an aggressive behavior, genomic instability, and present mutations in *BRCA1, BRCA2* and *TP53* genes [8]. 

Reportedly, the deletion or loss-of-function of single tumor suppressor genes associates with cancer progression and worse prognosis. However, conflicting data are present in the literature, which prompted us to undertake the present study. 

*TP53* tumor suppressor gene located on chromosome 17pl3 encodes a transcription factor for genes involved in the regulation of cell cycle, DNA repair, cell death and autophagy [9,10,11]. Mutations in *TP53* have been reported in approximately 40–80% of EOC [12,13,14], more frequently in advanced stage III and IV, and this correlated with worse clinical outcome [15,16,17]. Yet, in the TCGA cohort it was found that ovarian cancers with wild-type *TP53* were more chemoresistant and consequently associated with a poorer clinical outcome than those with mutated *TP53* [18]. 

*BRCA1* is one breast and ovarian cancer susceptibility gene that plays an important role in DNA damage repair and cell cycle regulation [19,20]. Germ-line mutation, somatic mutations, and methylation have been reported to alter *BRCA1* expression [21,22]. In advanced stage HGSOC, patients with germline *BRCA1/2* mutations have better prognosis [23,24,25]. *BRCA2* mutation, but not *BRCA1* mutation, was associated with significantly improved overall survival rate [26]. *BRCA1* negatively affects pro-survival autophagy in ovarian cancer cells [27,28]. 

*BECN1*, the first identified mammalian autophagy gene [29,30], maps on chromosome 17q21 in a region of around 150 kb centromeric to *BRCA1* [31]. Due to their close proximity, these two tumor suppressor genes are commonly co-deleted in breast and ovarian cancers and are expressed at reduced levels in these tumors [29,32]. BECLIN 1 regulates apoptosis by recruiting the anti-apoptotic protein of BCL-2 family (BCL-2 and BCL-XL) to the BH3 domain [33,34]. High expression of BECLIN 1 along with low expression of BCL-2 associates with good prognosis in non-Hodgkin lymphomas [35] and, conversely, low expression of *BECLIN 1* with high expression of *BCL-xL* associated with poor prognosis in hepatocellular carcinomas [36]. However, in nasopharyngeal carcinomas high level of BECLIN 1 along with HIF-1α predicts poor prognosis [37]. *BECN1* expression was found higher in benign and borderline ovarian tumors than in malignant EOC, suggesting that its decreased expression could favor tumorigenesis [38]. Accordingly, monoallelic loss of *BECN1* drives chromosome instability (leading to copy-number variation), increases migration, and promotes early ovarian tumors [39]. In an advanced stage of ovarian cancer, low expression of BECN1 and high level of BCL-2 (anti-apoptotic protein) was associated with poor prognosis [40,41]. Type I EOC was found to express higher levels of BECN1 and LC3 (the latter is an autophagy marker) compared to the aggressive Type II, and this was associated with chemo-responsiveness and better prognosis [42]. On the other hand, increased expression of *BECN1* was found to be associated with aggressive endometrioid adenocarcinomas and correlated with poor 5-years overall survival [43].

Thus, when considered individually, the altered expression of either of these three tumor suppressor genes appears associated with chemoresistance and poor clinical outcome or alternatively with chemosensitivity and good prognosis in ovarian cancer. Expectedly, the combined deletion or mutation of several tumor suppressor genes aggravates cancer progression and chemoresistance, thus worsening the prognosis. However, this assumption has not been validated yet for *BECN1*, *BRCA1* and *TP53* in EOC, three tumor suppressor genes that control autophagy, apoptosis, and DNA repair, and influence cancer cell fate in response to chemotherapy. 

Here, we have interrogated datasets from the TCGA database publicly available to better understand the role of *BECN1* and *BRCA1* deficiency in progression and chemo-responsiveness to platinum-based therapy in wild-type and mutated *TP53* ovarian cancers. Surprisingly, we found that the combined low/dysfunctional expression of these three main tumor suppressor genes in EOC associates with better prognosis, and this correlates with high responsiveness to platinum therapy. 

This study confirms that genotyping the cancer for certain critical oncogenes or tumor suppressor genes is mandatory for predicting the responsivity to chemotherapy, a step toward personalized treatments. Further, our study suggests that targeting the expression/function of both *BRCA1* and *BECN1* can improve the cytotoxic efficacy of platinum-based drugs. 

## 2. Methods

### 2.1. Genetic Profiling of Ovarian Serous Cystadenocarcinoma

Data of 316 patients, diagnosed for ovarian serous cystadenocarcinoma, were retrieved from The Cancer Genome Atlas (TCGA) (https://portal.gdc.cancer.gov/, accessed on 27 October 2020) [14]. The patients’ characteristics have been reported in Supplementary Table S1, and the selection and exclusion criteria of the study cohort is depicted in the flow chart shown in Supplementary Figure S1. All the patients underwent primary cytoreductive surgery, followed by an adjuvant platinum-based chemotherapy as a first-line treatment. Eligibility criteria for the study were (1) histologically verified diagnosis of high-grade serous ovarian adenocarcinomas; (2) lack of neo-adjuvant therapy; (3) platinum sensitivity (the time from adjuvant platinum-based treatment to cancer relapse (platinum-free interval (PFI), was >6 months).

In TCGA ovarian cancer mRNA gene expression, copy number variation (CNV), *TP53*-mutation status, DNA methylation and clinical data (FIGO stage, grade, overall survival and disease-free status and platinum sensitivity/resistance) were downloaded from the cBioportal.org. 

TCGA gene expression profile was measured using the Illumina HiSeq 2000 RNA Sequencing platform (Illumina Inc., 9885 Towne Centre Drive, San Diego, CA 92121, USA). RSEM (RNA-Seq by Expectation-Maximization) normalized count was used as gene level expression estimates. TCGA copy number profile was measured using genome-wide SNP6 array. Gene-level somatic copy number alterations were estimated using the GISTIC 2.0 [44] method which summarized the copy number of each gene into −2, −1, 0, 1, 2, representing the homozygous/deep deletion (−2), heterozygous/shallow deletion (−1), diploid normal copy (0), low-level amplification (+1), or high-level amplification (+2), respectively (Supplementary Table S1). *TP53*-mutation status was also obtained from the TCGA data portal (Supplementary Table S1). Lastly, TCGA tumor grade and stage information were manually extracted from the pathologic reports provided by the cBioportal.

### 2.2. Statistical Analysis

The analysis focused on 316 ovarian serous cystadenocarcinoma in TCGA dataset. For statistical significance, the analysis was performed on tumors with shallow deletion and diploid CNV that were the most represented ones.

*BECN1* and *BRCA1* were grouped based on (i) the CNV (copy number variation) and (ii) the level of mRNA expression in ovarian cancer patients. The latter was sub-classified based on the level of Z-score values as high, medium and low, respectively. Low *versus* high mRNA expression was defined relative to the median expression level of all patients in the form of a box plot and were used to investigate the relationship between dichotomized *BECN1* and *BRCA1* expression. To reduce the potential bias from dichotomization, the mRNA expression of *BECN1* and *BRCA1* were compared based on CNV, *TP53* mutation and expression-based groups using t-test (Welch Two Sample *t*-test) by R. All cut-off values were set before the analysis, and all the tests were two-tailed.

All statistical analyses were performed by R (3.6.1 version, The R Foundation for Statistical Computing, Vienna, Austria) and SAS software (9.4. version, SAS Institute Inc., Cary, NC, USA) using SAS/STATs procedures for *BECN1* and *BRCA1* in different sample sized groups. 

Survival analysis was performed using SAS for the following: *TP53* mutation, CNV and mRNA expression level-based groups of *BECN1* and *BRCA1*. Survival curves of these three groups were estimated by the Kaplan–Meier plots and compared using the Cox regression model assuming an ordered trend for the three groups as described previously. The log-rank test has been used to determine the statistical significance. The *p*-value < 0.05 was considered to be significant.

## 3. Results

### 3.1. Clinical Characteristics of the Patient Cohort 

The Ovarian Cancer Data Set (Consortium TCGA, Nature 2011) available in TCGA database (www.cBioportal.org, accessed on 27 October 2020) consists of 316 High Grade Serous Ovarian Cancer patients (HGSOC). The cohort of patients with HGSOC comprised cases with N/A (1) II (14), III (248), and IV (53) FIGO stages. The tumor grade was determined as G2 in 28 cases, G3 in 281 cases, and undetermined for other cases. All patients underwent primary cytoreductive surgery, followed by first-line platinum-based chemotherapy. All patients experienced a complete or a partial response post-adjuvant therapy with platinum-free interval (PFI) >6 months. Patients reporting data on *TP53* (mutated and wild type), *BECN1* and *BRCA1*, were categorized based on putative changes in DNA copies (CNV). Diploid, shallow deletion, amplification and gain of *BECN1* and *BRCA1* genes and clinical data are detailed in Supplementary Table S1

### 3.2. Oncoprint of Somatic Mutations in Ovarian Cancer

**Figure 1:**
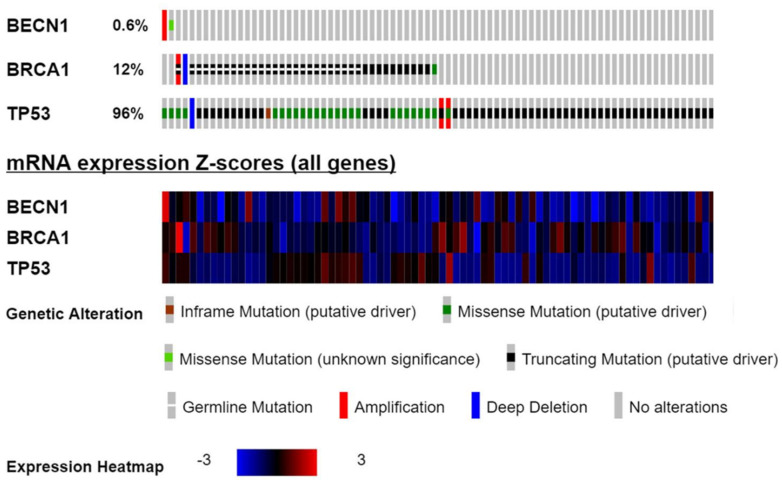
The oncoprint showing the genetic alterations and mRNA expression level of *BECN1*, *BRCA1* and *TP53* in 316 ovarian serous cystadenocarcinomas from the TCGA dataset (Consortium TCGA, Nature 2011).

As represented in Figure 1, in a total of 316 ovarian cancers, *BECN1* has been observed amplified in one patient and bearing a missense mutation in another patient (0.6%); *BRCA1* mutated cases were observed in 38 patients (12%), and mutated *TP53* was reported in as many as 303 patients (96%). As for the type of genetic alteration, missense and truncating mutations were the most frequent ones. Figure 1 also reports the level of mRNA expression of the three tumor suppressor genes. It is evident that with a few exceptions the large majority of the EOC analyzed express low or very low level of mRNA for the three tumor suppressor genes, especially in the case of *BECN1*, while abnormally high level of *BECN1* or *BRCA1* mRNA is observed in only one case, respectively, that associates with gene amplification. 

### 3.3. Patients with Mutated TP53 Have Significantly Better Survival Than Those with Wild Type TP53

**Figure 2:**
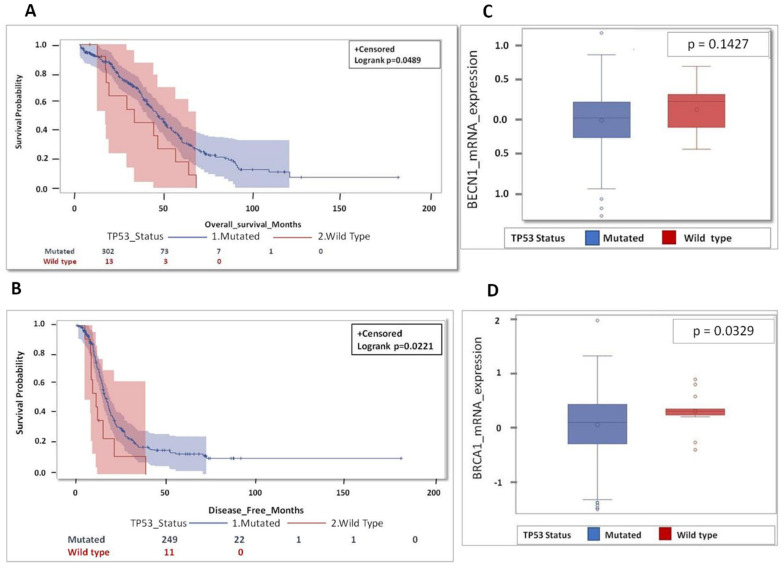
Patients bearing a *TP53*-mutated tumor show better prognosis compared to *TP53* wild type. (**A**) Overall survival for HGSOC patients according *TP53* status. (**B**) Disease-free survival (DFS) for HGSOC patients according *TP53* status. (**C**) Box-plot showing the distribution of *BECN1* expression according *TP53* status. (**D**) Box-plot showing the distribution of *BRCA1* expression according *TP53* status. (Note that for OS data were not available for 1 patient (mutated group 1 pt: 302 pt’s total *n* = 303 mutated pt’s) while for DFS data were missing for 56 patients (mutated group 54 pt: 249 pt’s+ wild type group 2 pt’s: 11 pt’s; total *n* = 316 pt’s), respectively).

First, we focused on *TP53* status. Patients with mutated *TP53* (*n* = 303) showed significantly better overall and disease-free survival with significant values *p* = 0.0489 and *p* = 0.0221 respectively, than those with wild type *TP53* (*n* = 13 and *n* = 11, respectively) (Figure 2A,B). The median overall survival among the patients with wild type *TP53* was 27 months, while it was about 50 months in mutated *TP53* patients. Intriguingly, the group of tumors with mutated *TP53* presented lower expression of *BECN1* (*p* = 0.1427) and of *BRCA1* (*p* = 0.0329) than the group of tumors with wild type *TP53* (Figure 2C,D). As these findings were unexpected, and somehow counterintuitive, we further interrogated the data base in search of a possible explanation for the poor prognosis in the wild-type *TP53* group (possibly due to additional oncogenic mutations) and for the good prognosis in mutated *TP53* group (possibly related to therapy responsiveness). 

### 3.4. Mutational Profiles of High-Grade Ovarian Serous Cystadenocarcinoma with Wild Type TP53

In the 13 tumor samples with wild type *TP53* from TCGA data (validated for somatic mutation status), we identified 490 genes that were mutated (Supplementary Table S2). This list of genes was analyzed by using DAVID bioinformatics functional annotation tool (https://david.ncifcrf.gov/summary.jsp, accessed on 22 September 2020) to obtain Gene Ontology (GO) biological processes. We found 267 genes involved in pathogenesis of cancer, which could have contributed to ovarian carcinogenesis despite the normally functioning of *TP53* (Supplementary Figure S2).

### 3.5. BECN1 and BRCA1 Deletions Correlate with High Tumor Stage and Grade

**Figure 3:**
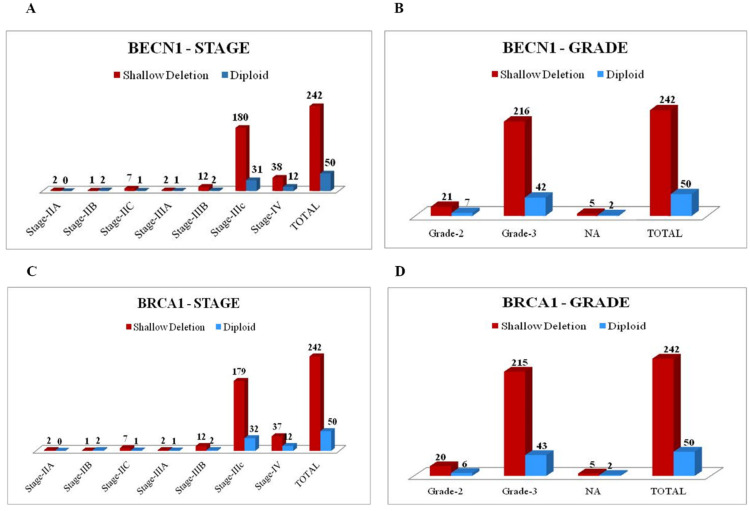
Shallow deletion of *BECN1* and *BRCA1* correlates with stage IIIC and grade 3 in ovarian cancers. (**A**) Graph representing the distribution of *BECN1* copy number variation (CNV) according to tumor stage. (**B**) Graph representing the distribution of *BECN1* CNV according to tumor grade. (**C**) Graph representing the distribution of *BRCA1* CNV according to tumor stage. (**D**) Graph representing the distribution of *BRCA1* CNV according to tumor grade.

Most patients were diagnosed with tumors at stage III and IV (78.4% and 16.8% respectively), and of grade G3 (88.9%). As shown in Figure 3, shallow co-deletion of *BECN1* and *BRCA1* occurs in >83% of the tumors at stage IIIc and IV and of grade 3. 

### 3.6. Correlation of BECN1 and BRCA1 Co-deletion

In the 316 ovarian cancers in TCGA, *BECN1* and *BRCA1* were shallow (monoallelic) deleted in 242 (76.6%) and 240 (76%) cases, respectively (Supplementary Table S1). A co-occurrence analysis of CNV revealed that the two events were highly correlated (Supplementary Table S3), which was expected given the close proximity of these two genes on chromosome 17q21. Out of 316 cases, CNV was identical for both *BECN1* and *BRCA1* in 313 cases (50 diploid, 240 shallow deletion, 23 amplification), while 2 cases with *BECN1* shallow deletion were associated one with amplification and one with deep deletion of *BRCA1*, and one case diploid for *BRCA1* was amplified for *BECN1* (Supplementary Table S3). It is to be noted that no one tumor had a deep (homozygous) co-deletion of both *BECN1* and *BRCA1*, and only one tumor had a deep deletion of *BRCA1* associated with shallow deletion of *BECN1*. 

### 3.7. Patients with Shallow Deletion of BECN1 and BRCA1 Have Better Prognosis

To determine the prognostic role of *BECN1* and *BRCA1*, we analyzed the survival of patients bearing a tumor with consistent CNV of the tumor suppressor genes, i.e., with both shallow deletion (240 cases) or diploid (50 cases) status (see Supplementary Table S3). Overall survival and disease-free survival data were not available for 1 (diploid) case and for 51 (16 diploid and 35 shallow deleted) cases, respectively. 

**Figure 4:**
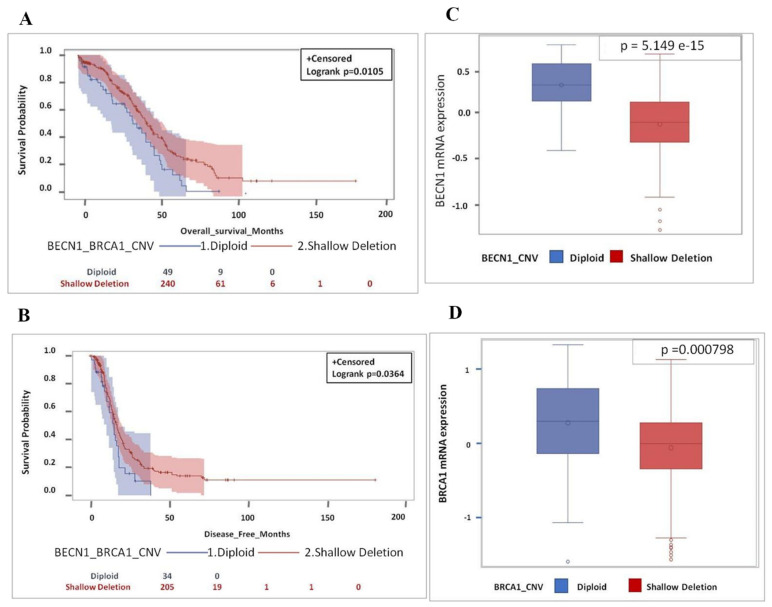
Shallow deletion of *BECN1* and *BRCA1* with low mRNA expression is associated with better overall and disease-free survival. (**A**) Overall survival for HGSOC patients according to *BECN1* and *BRCA1* CNV. (**B**) Disease-free survival for HGSOC patients according to *BECN1* and *BRCA1* CNV. (**C**) Box-plot showing the distribution of *BECN1* expression according to CNV. (**D**) Box-plot showing the distribution of *BRCA1* expression according to CNV. (Note that for OS data were not available for 1 patient (diploid group - 1 pt: 49 pt’s; diploid group total *n* = 50 pt’s) while for DFS data were missing for 51 patients (diploid group - 16 pt’s: 34 pt’s + shallow deletion group - 35 pt’s: 205 pt’s; total *n* = 290 pt’s), respectively).

Based on *BECN1* and *BRCA1* CNV (diploid versus shallow deletion), it is apparent that patients bearing an ovarian cancer with shallow deletion of the two tumor suppressor genes have a significant better prognosis, i.e., overall and disease-free survival (*p* = 0.0105 and *p* = 0.0364) than the patients bearing a cancer with diploid CNV, independently of the *TP53* status (Figure 4A,B). We confirmed that shallow deletion corresponded to lower expression of the gene. As shown in Figure 4C,D, the average level of mRNA expression of *BECN1* and *BRCA1* in the 240 tumors with shallow deletion CNV was lower than the corresponding level in the 50 tumors with diploid CNV, indicating that the monoallelic loss of the tumor suppressor gene reflected in lower mRNA expression. Notably, the relationship between copy number loss and mRNA expression was more significant for *BECN1* than for *BRCA1* in this dataset (*p*-value = 5.149 × 10^−15^ and *p*-value = 0.000798, respectively).

### 3.8. Patients Bearing a Tumor with Shallow Co-deletion of BECN1 and BRCA1 along with Mutated TP53 Have Better Prognosis

*TP53* was mutated in 43 (86%) of 50 cases diploid for both *BECN1* and *BRCA1*, and in 236 (98%) of the 240 cases shallow deleted for *BECN1* and *BRCA1*. The low number (*n* = 11; 7 CNV diploid + 4 CNV shallow for *BECN1* and *BRCA1*) of patients with wild type *TP53* does not allow to draw conclusion statistically significant. Since *TP53* was mutated in the vast majority of the tumors, we asked about its contribution in the prognostic value of *BECN1* and *BRCA1*. 

**Figure 5:**
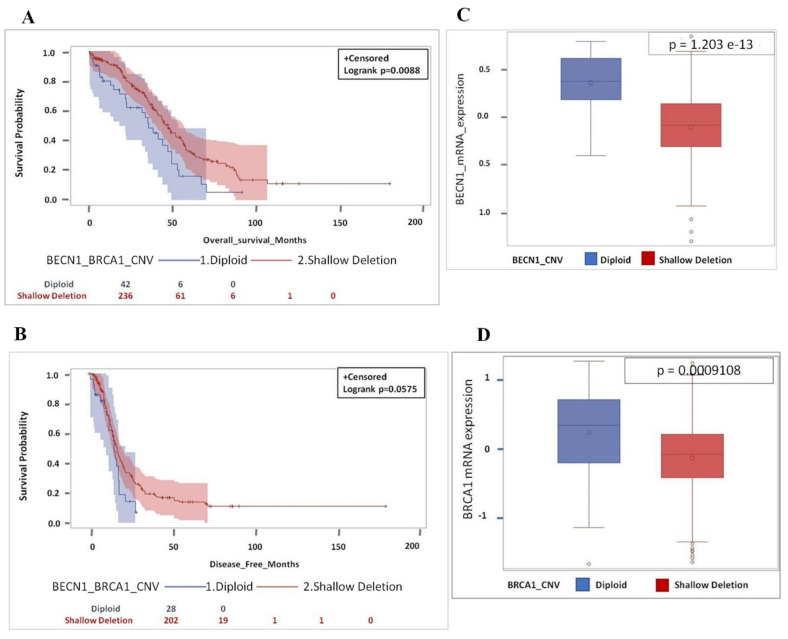
Shallow deletion of *BECN1* and *BRCA1* with low mRNA expression in *TP53*-mutated patients is associated with better overall survival as well as disease-free survival. (**A**) Overall survival for HGSOC patients based on *BECN1* and *BRCA1* CNV. (**B**) Disease-free survival for HGSOC patients based on *BECN1* and *BRCA1* CNV. (**C**) Box-plot showing the distribution of *BECN1* expression according to CNV. (**D**) Box-plot showing the distribution of *BRCA1* expression according to CNV. (Note that for OS data were not available for 1 patient (diploid group 1 pt: 42 pt’s; diploid group total *n* = 43 pt’s) while for DFS data were missing for 49 patients (diploid group 15 pt’s: 28 pt’s + shallow deletion group 34 pt’s: 202 pt’s; total *n* = 279 pt’s), respectively).

As expected, overall survival (*p* = 0.0088) and disease-free survival (*p* = 0.0575) were higher in the patients bearing a tumor with shallow deletion of both *BECN1* and *BRCA1* compared to those bearing a tumor with a diploid CNV of the two tumor suppressor genes (Figure 5A,B). Notably, the *p*-value of the overall survival of the patients bearing cancer with shallow CNV of *BECN1* and *BRCA1* was more significant in *TP53* mutated than in the whole cohort (*p* = 0.0088 versus *p* = 0.0105) (Figure 4A and Figure 5A). It was also confirmed that copy number loss (shallow deletion) of *BECN1* and *BRCA1* corresponded to lower level of the respective mRNA (Figure 5C,D; *p* = 1.203 × 10^−13^ and *p* = 0.0009108, respectively). 

### 3.9. Low Level of BECN1 and BRCA1 mRNA in TP53-mutated Ovarian Cancer Predicts Better Patient Prognosis

Previous data showed that shallow CNV of *BECN1* and *BRCA1* correlates with low level of mRNA in the tumor and with better prognosis of the patients. However, gene expression rather than gene copy number defines the phenotype. Low gene expression depends also on epigenetic silencing, besides gene copy number loss. One important epigenetic modification is the DNA methylation (particularly, the 5-methylcytosine within CpG isles) in the gene promoter, which can be detected with the HumanMethylation27 (HM27k) array [45]. We found that decreased expression of *BECN1* and *BRCA1* in ovarian cancers was significantly associated with high DNA methylation of these genes (Supplementary Figure S3). 

**Figure 6:**
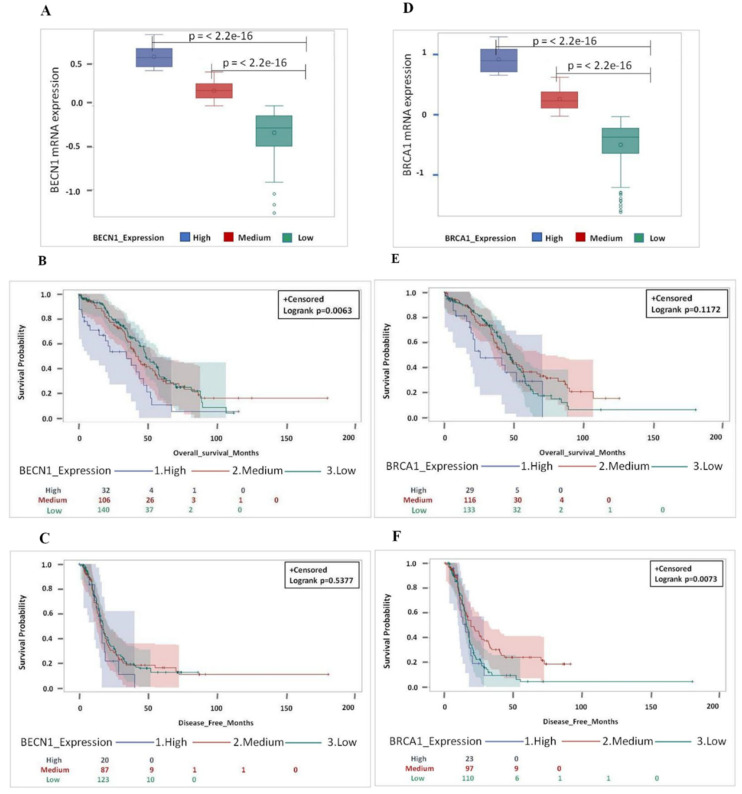
Low expression of both *BECN1* and *BRCA1* independently gives better and longer overall survival as well as disease-free survival in *TP53*-mutated patients. (**A**) Box-plot showing the distribution of *BECN1* expression based on expression levels (high, medium and low). (**B**) Overall and (**C**) disease-free survival for HGSOC patients based on *BECN1* expression levels (high, medium and low). (**D**) Box-plot showing the distribution of *BRCA1* expression based on expression levels (high, medium and low). (**E**) Overall and (**F**) disease-free survival analysis for HGSOC patients based on *BRCA1* expression levels (high, medium and low). (Note that for *BECN1* OS data were not available for 1 patient (medium group 1 pt: 106 pt’s; medium group total *n* = 107 pt’s) while for *BECN1*—DFS data were missing for 49 patients (high group 12 pt’s: 20 pt’s + medium group 20 pt’s: 87 pt’s + low group 17 pt’s: 123 pt’s; total *n* = 279 pt’s), respectively. For *BRCA1* - OS data were not available for 1 patient (low group 1 pt: 133 pt’s; low group total *n* = 134 pt’s) while for *BRCA1*-DFS data were missing for 49 patients (high group 6 pt’s: 23 pt’s + medium group 19 pt’s: 97 pt’s + low group 24 pt’s: 110 pt’s; total *n* = 279 pt’s), respectively).

We have classified the tumors for the expression level of *BECN1* and *BRCA1* mRNA in three distinct groups (high, medium, and low) according to the z-score values and the methylation status, as indicated in Supplementary Figure S3. Next, we looked at the prognosis in patients bearing a *TP53*-mutated ovarian cancer according to the high or medium or low level of mRNA expression of *BECN1* and *BRCA1* (Figure 6). Patients with the medium or low level of *BECN1* and *BRCA1* expression (corresponding to a high methylation profile) showed a significant better prognosis (i.e., long overall survival and disease-free survival) than the patients bearing tumors with high level of *BECN1* and *BRCA1* mRNAs (Figure 6).

We classified the patients with medium and low level of mRNA expression of the two genes as low expressors and found that, compared to this group of patients, the group of high expressors showed poor prognosis (Supplementary Figure S4).

**Figure 7:**
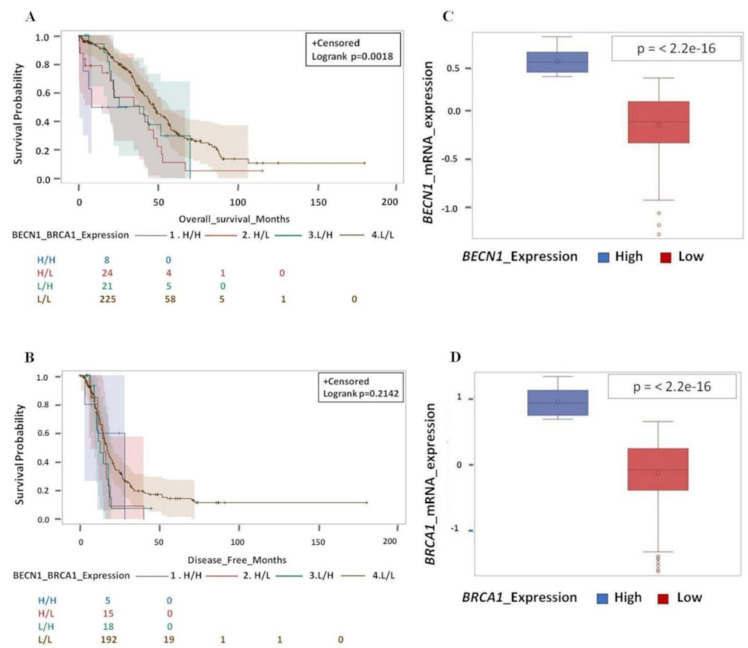
Low expression of both *BECN1* and *BRCA1* in conjugation gives better and longer overall survival as well as disease-free survival. (**A**) Overall survival for HGSOC patients according to *BECN1* and *BRCA1* expression level (H/H, H/L, L/H and L/L). (**B**) Disease-free survival for HGSOC patients according to *BECN1* and *BRCA1* expression level (H/H, H/L, L/H and L/L). (**C**) Box-plot showing the distribution of *BECN1* according to expression level (high and low). (**D**) Box-plot showing the distribution of *BRCA1* according to expression level (high and low). (Note that for OS data were not available for 1 patient (L/L group 1 pt: 225 pt’s; L/L group total *n* = 226 pt’s) while for DFS data were missing for 49 patients (H/H group 3 pt’s: 5 + H/L group 10 pt’s: 15 pt’s + L/H group 6 pt’s: 18 pt’s + L/L group 34 pt’s: 192; total *n* = 279 pt’s), respectively).

Next, we attempted to discriminate the individual contribution of *BECN1* and *BRCA1* expression to the prognosis. To this end, we grouped the cases with medium and low expression of each single gene and analyzed the prognostic value of the combinatorial groups of tumors based on the respective level of mRNA expression of *BECN1* and *BRCA1* as follow: H/H (High *BECN1*/High *BRCA1*), H/L (High *BECN1*/Low *BRCA1*), L/H (Low *BECN1*/High *BRCA1*), and L/L (Low *BECN1*/Low *BRCA1*). From this analysis it appears evident that tumors with high mRNA expression of both genes (H/H) have the worse prognosis, while those with low mRNA expression of both genes (L/L) have the best prognosis (Figure 7). On the other hand, the individual lower expression of either *BECN1* or *BRCA1* (the combination L/H or H/L) does not confer significant improvement of the clinical outcome, suggesting that it is the concurrent loss of function of both the tumor suppressor genes that determines the good prognosis. 

### 3.10. BECN1 and BRCA1 Deficiency Correlates with Platinum Sensitivity 

**Figure 8:**
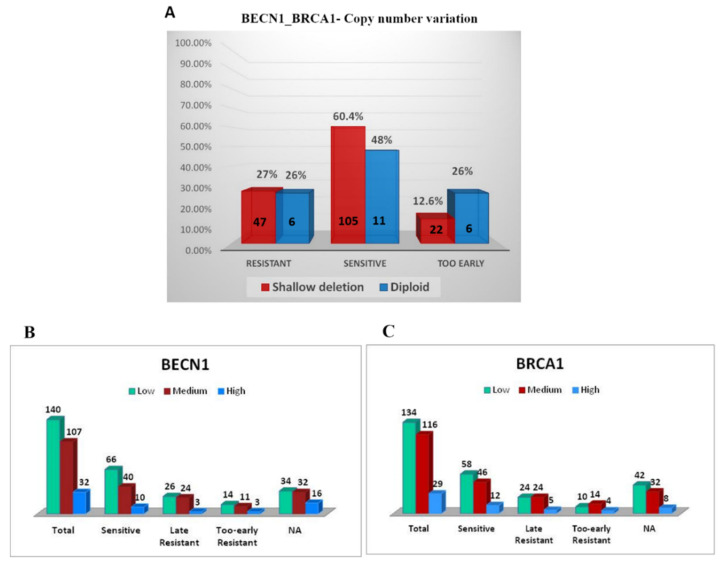
Concurrent low expression of *BECN1* and *BRCA1* correlates with platinum sensitivity. (**A**) Platinum sensitivity based on CNV of *BECN1* and *BRCA1* in *TP53*-mutated patients. (**B**) Platinum sensitivity based on *BECN1* mRNA expression. (**C**) Platinum sensitivity based on *BRCA1* mRNA expression. All patients underwent primary cytoreductive surgery, followed by adjuvant platinum-based chemotherapy as first-line treatment. Platinum sensitivity: the time from adjuvant platinum-based treatment to cancer relapse (platinum-free interval, PFI) was >6 months. Late Resistance: resistance occurs after a 6-month-period. Too-early Resistance: resistance occurs as soon as treatment started (within 6 months).

Platinum resistant patients show disease progression within six months of primary therapy, while platinum sensitive patients show relapse or disease progression six months after the end of treatment. From a total of thirteen wild type *TP53* patients, only three were found chemo-sensitive and eleven were deceased. Of the latter, five were resistant to the standard platinum chemotherapy and have recurred the disease in less than 6 months. Unfortunately, data regarding other patients were not available (N/A) (Supplementary Table S4). Taken together, above data indicate that the patients bearing a tumor with wild type *TP53* experience bad prognosis, and this associates with high expression of *BECN1* and *BRCA1* mRNA and with platinum-resistance, while the opposite is observed for the patients bearing a tumor with mutated *TP53* that associated with decreased mRNA levels of *BECN1* and *BRCA1* (Figure 1). To be noted, of 213 EOC with mutant *TP53*, 125 (58.69%) are sensitive, 57 (26.76%) are late resistance and 31 (14.55%) are too-early resistant (for 90 patients’ data were not available (N/A)). Therefore, we asked about the response toward the platinum-based chemotherapy in the *TP53*- mutated tumors with respect to the combined *BECN1* and *BRCA1* copy number variation (279 cases). To be noted, for 82 of these patients (approx. 30%; 62 with shallow deletion and 20 diploid) no information on the platinum sensitivity in relation to *BECN1* and *BRCA1* CNV status was available. Of the patients bearing a tumor with *BECN1* and *BRCA1* shallow deletion (174), 105 (60.4%) of patients were sensitive, 47 (27%) were resistant and 22 (12.6%) developed early resistance. In contrast, of 23 patients bearing a tumor with diploid CNV of *BECN1* and *BRCA1* only 11 (48%) were sensitive, 6 (26%) developed resistance and 6 (26%) developed an early resistance, as shown in Figure 8. 

Finally, platinum-responsive status (116 patients) was correlated to low, medium and high expression of *BECN1* and *BRCA1*. Patients with low levels of *BECN1* (66 patients) were significantly more sensitive compared to medium *BECN1* expressors (40 patients), while only 10 patients with high *BECN1* expression were sensitive to the therapy. A similar trend was observed for *BRCA1* expression. Both low (58 patients) and medium (46 patients) *BRCA1* mRNA expressors were sensitive to platinum treatment, whereas only 12 patients with high *BRCA1* expression manifest a great response to chemotherapy. 

Because of the small number of patients, we could not demonstrate a significant association between the high expression of *BECN1* and *BRCA1* genes and chemosensitivity, however the collected data indicate that high expression of both these tumor suppressor genes results in chemotherapy resistance while medium-low expression is significantly associated with platinum-sensitivity.

## 4. Discussion

The stratification of HGSOC patients in poor and good responders to the platinum-based first-line therapy is crucial for implementation of personalized ovarian cancer treatment, and for this it is important to genotyping the tumor for critical risk genes [46,47]. Dysfunction of tumor suppressor genes (low expression or loss-of-function mutations) has been associated with tumor development and progression, and with poor clinical outcome. However, the contribution of one single risk gene is influenced by the concurrent mutation of other risk genes involved in the same or intersected processes [46], and this is likely to affect the final outcome.

Here, we focused on the concurrent contribution of three main tumor suppressor genes, namely *TP53*, *BECN1* and *BRCA1*, in determining the clinical response to therapy and clinical outcome in ovarian cancer patients. These genes were chosen because of their primary role in the control of the pathways that govern cell survival, cell death and DNA repair, and ultimately the response to chemotherapeutic injury. These three tumor suppressor genes locate on chromosome 17, and are frequently found co-mutated [32,48]. Consistently, in the 316 ovarian cancers present in the TCGA, as many as 236 were showing deletion or mutation in these three tumor suppressor genes.

In our first analysis we found that 13 patients bearing a wild type *TP53* HGSOC had worse prognosis associated with chemoresistance compared to the 303 patients bearing a mutated *TP53* tumor. This is a confirmation of the previous study by Wong et al (2013) on the same database [18]. Note that more recently the number of wild type *TP53* cases has been revised from 15 to 13 [13]. Compared to that study where 88 genes involved in carcinogenesis were found mutated [18], we have updated the list of the mutated genes (260 out of 490) that could have contributed to the bad prognosis in the patients with wild type *TP53* (Supplementary Table S2). These genes appear mainly involved in cell receptor signaling, cell morphogenesis and cell adhesion and migration (Supplementary Figure S2).

Most importantly, and of relevance for our aim, we found that tumors with wild type *TP53* had a tendency to express higher level of *BRCA1* (statistically significant) and of *BECN1* (though not significant) than the tumors with mutated *TP53*. Low mRNA expression of *BECN1* and *BRCA1* was confirmed in the subset of tumors with mutated *TP53*, and it was significantly correlated with platinum sensitivity and good prognosis. We found that low mRNA expression of both genes arose from monoallelic (shallow) deletion as well as from promoter hyper-methylation. Monoallelic co-deletion of *BECN1* and *BRCA1* has been reported in 40-75% of sporadic ovarian cancers [31]. *BECN1* is a haploinsufficient tumor suppressor gene, since it opposes tumorigenesis when over-expressed [29] but favors tumorigenesis when present in single copy (30). *BRCA1* also is *bona fide* a haploinsufficient tumor suppressor gene, though its heterozygous expression does not promote spontaneous tumors [49]. We observed that the vast majority of the tumors at stage III and IV and of grade 3 were bearing shallow deletions (i.e., expressing one single allele) of both *BECN1* and *BRCA1*, further indicating that the low expression of these tumor suppressor genes associates with ovarian cancer progression. Accordingly, the expression of *BECN1* was found significantly higher in benign and borderline ovarian cancer than in aggressive ovarian cancer, and inversely correlated with the increasing FIGO stage and histologic grade of malignant EOC [38]. Yet, another study found that the expression of BECLIN 1 protein was stronger in ovarian carcinoma than that in normal ovary and benign tumor [50]. It was also found that EOC expressed higher level of BECLIN 1 along with lower level of BRCA1 in EOC than in benign epithelial ovarian tumor tissues [51]. Our results are coherent with previous findings showing cisplatin sensitivity in ovarian cancers with low expression of *BRCA1* [51,52,53]. Interestingly, low expression of BECLIN 1 and of BRCA1 proteins identified platinum-responsive patients, and this was associated with impaired autophagy [51]. Consistently, *BRCA1* deficient ovarian cancer cells were highly dependent on BECLIN 1-dependent autophagy for survival, and rapidly underwent cell death upon autophagy disruption [27,28]. In summary, the present study demonstrates that the concurrent low expression of *BECN1* and *BRCA1* is sufficient to make ovarian cancer cells more sensitive to chemotherapeutics. In principle, it might seem counterintuitive that low expression of two tumor suppressor genes favors a good clinical outcome. This is particularly surprising since both *BECN1* and *BRCA1* were found expressed at very low level in the tumors with more aggressive phenotype (stage and grade). However, what finally determines if the cell will survive or succumb to a cytotoxic stress much depends on the combination of the risk genes that loose or gain in function and how this combination impact on the pathways controlling autophagy, cell death, DNA repair and cell metabolism. BRCA1 deficiency was shown to promote BECLIN 1-dependent autophagy that protected ovarian cancer cells (and benign ovarian epithelial cells) from toxic metabolic stress [27]. Thus, the loss of *BECN1* combined with *BRCA1* deficiency probably compromises autophagy-mediated protection from platinum-toxicity. In this respect, it is worth noting that no one tumor had a deep (homozygous) co-deletion of both *BECN1* and *BRCA1*, which is consistent with the fact that complete loss of these tumor suppressor genes is incompatible with cell survival [30,54]. As a matter of fact, our analysis shows that the down-regulation of *BECN1* and *BRCA1* expression could restore the ability of ovarian cancer cells to respond to platinum-based therapy, even in wild-type *TP53* patients. Translating into clinics the above considerations, one could envisage a therapeutic strategy for knocking-down both *BRCA1* and *BECN1* to drive ovarian cancer cell death. In this respect, phytochemicals acting as epigenetic modulators could represent a source of valid adjuvant therapeutics [55,56].

## 5. Conclusions

We have interrogated datasets from the TCGA database to define how the genetic and epigenetic alterations of *BECN1*, *BRCA1* and *TP53*, three tumor suppressor genes that control autophagy, apoptosis, and DNA repair, contribute to the chemo-responsiveness to platinum-based therapy and the prognosis in ovarian cancers. Our analysis demonstrates that the concurrent low expression of *BECN1* and *BRCA1* is sufficient to make ovarian cancer cells more sensitive to chemotherapeutics. The present study confirms the importance of genotyping the cancer for critical risk genes involved in chemoresistance for designing a personalized therapy. 

## Data Availability

The data that support the findings of this study are available from the corresponding author upon reasonable request.

## Supplementary Materials

The following are available online at https://www.mdpi.com/2227-9059/9/2/207/s1. Supplementary Figure S1: Flow diagrams depicting the selection of the study population; Supplementary Figure S2: Biological processes associated with mutated genes in 13 patients bearing an ovarian cancer with wild type *TP53*; Supplementary Figure S3: Correlation of *BECN1* and *BRCA1* mRNA expression with methylation values of their respective gene; Supplementary Figure S4: High expression of *BECN1* and *BRCA1* gives poor overall and disease-free survival compared to low plus medium *BECN1* and *BRCA1* mRNA expression in *TP53*-mutated patients; Supplementary Table S1: Characteristics of High Grade Serous Ovarian Cancer patients in TCGA database; Supplementary Table S2: Gene mutations in TCGA ovarian samples with wild type *TP53*; Supplementary Table S3: Correlation between CNV of *BECN1* and *BRCA1*; Supplementary Table S4: Clinical outcome of patients bearing the ovarian tumor with wild type *TP53*.
