# Supplementary material for: BECN1 and BRCA1 Deficiency Sensitizes Ovarian Cancer to Platinum Therapy and Confers Better Prognosis
Source: Biomedicines. 2021 Feb 18;9(2):207. doi: 10.3390/biomedicines9020207 (PMC7922320; doi:10.3390/biomedicines9020207)
Supplement: Supplementary file 1 [file biomedicines-09-00207-s001.zip › biomedicines-1068460-supp/Supplementary Figures.docx]

**
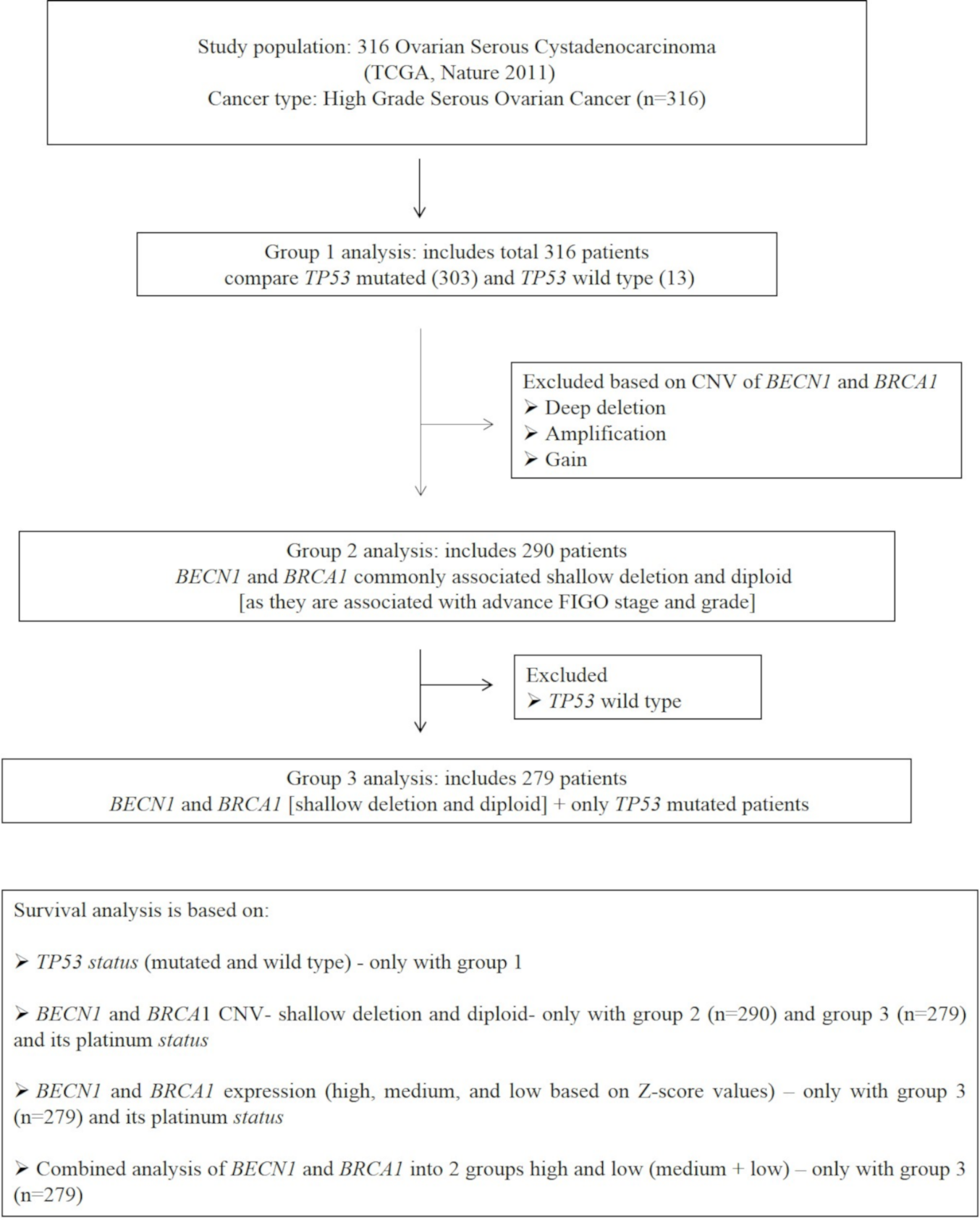
**

**Supplementary Figure S1. Flow diagrams depicting the selection of the study population.**


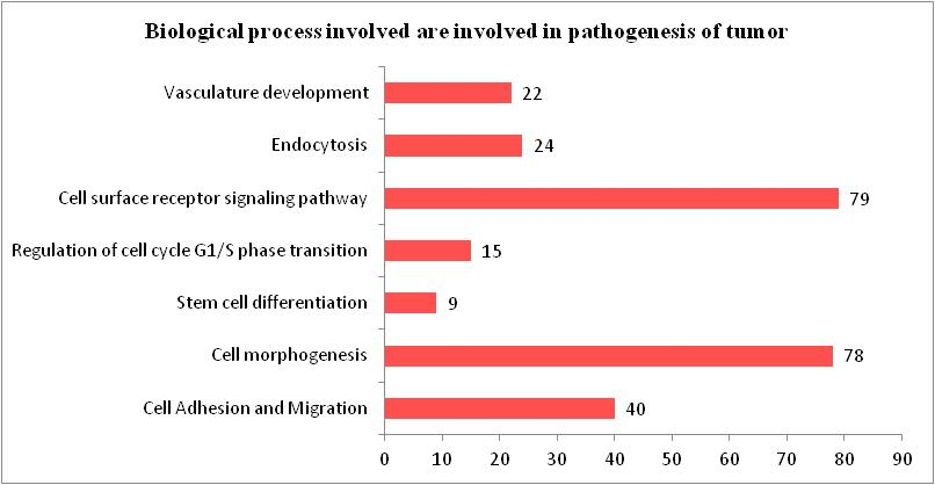


**Supplementary Figure S2. Biological processes associated with mutated genes in 13 patients bearing an ovarian cancer with wild type *TP53*.**


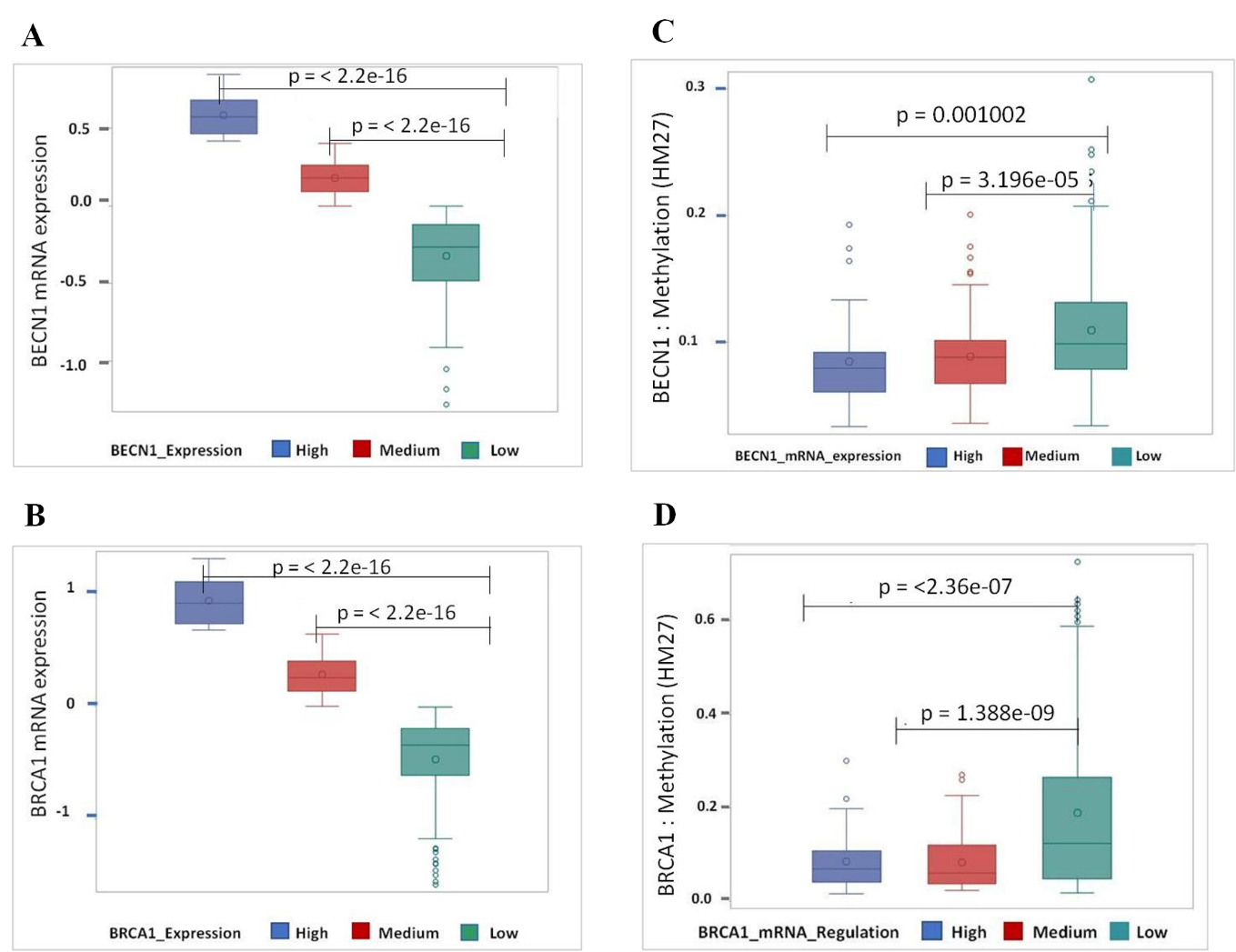


**Supplementary Figure S3. Correlation of *BECN1* and *BRCA1* mRNA expression with methylation values of their respective gene.** A) Box-plot showing the distribution of *BECN1* based on expression levels (high, medium and low). B) Box-plot showing the distribution of *BRCA1* based on expression levels (high, medium and low). C) Distribution of *BECN1* methylation based on mRNA expression levels (high, medium and low). D) Distribution of *BRCA1* methylation based on mRNA expression levels (high, medium and low).

**
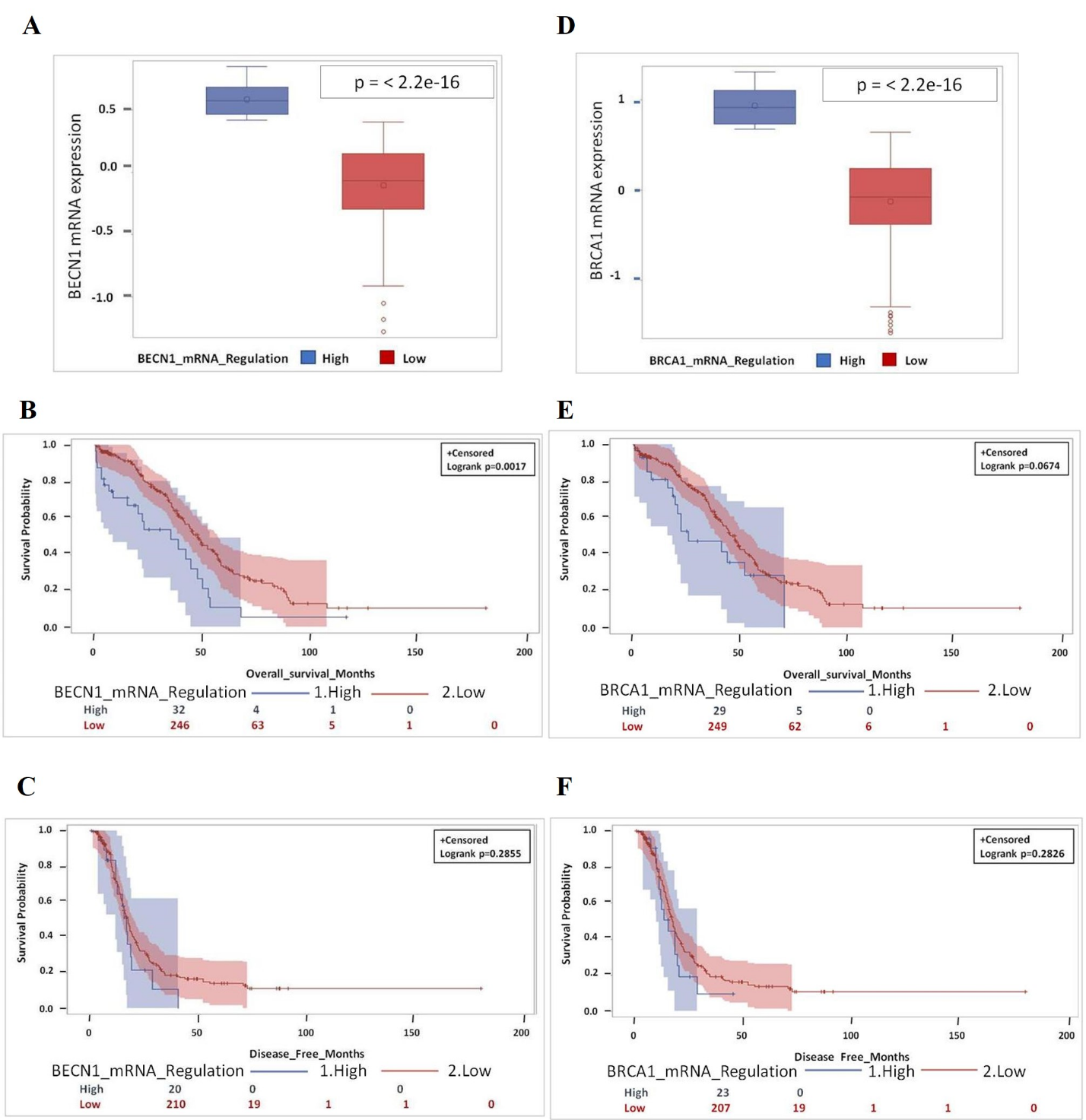
**

**Supplementary Figure S4. High expression of *BECN1* and *BRCA1* gives poor overall and disease-free survival compared to low plus medium *BECN1* and *BRCA1* mRNA expression in *TP53*-mutated patients.** A) Box-plot showing the distribution of *BECN1* based on expression levels (high and medium plus low). B-C) Overall (B) and disease-free (C) survival for HGSOC patients based on *BECN1* expression levels (high and medium plus low). D) Box-plot showing the distribution of *BRCA1* based on expression levels (high and medium plus low). E-F) Overall (E) and disease-free (F) survival analysis for HGSOC patients based on *BRCA1* expression levels (high and medium plus low). (Note that for *BECN1* - OS data were not available for 1 patient (low group 1 pt: 246; low group total n=247 pt’s) while for *BECN1* - DFS data were missing for 49 patients (high 12 pts: 20 + low 37 pt’s: 210 pt’s; total n=279 pt’s), respectively. For *BRCA1* - OS data were not available for 1 patient (1 pt low group: 249; total n=250 pt’s low group) while for *BRCA1* - DFS data were missing for 49 patients (high 6 pts: 23 + low 43 pt’s: 207 pt’s; total n=279 pt’s), respectively).
