# Supplementary material for: BECN1 and BRCA1 Deficiency Sensitizes Ovarian Cancer to Platinum Therapy and Confers Better Prognosis
Source: Biomedicines. 2021 Feb 18;9(2):207. doi: 10.3390/biomedicines9020207 (PMC7922320; doi:10.3390/biomedicines9020207)
Supplement: Supplementary file 1 [file biomedicines-09-00207-s001.zip › biomedicines-1068460-supp/Supplementary Tables.docx]

**Supplementary Table S1. Characteristics of High Grade Serous Ovarian Cancer patients in TCGA database.**

| Clinical Characteristics’ | Number | Percentage |
| --- | --- | --- |
| High Grade Serous Ovarian Cancer | 316 | 100 |
| Tumor Residual Disease  1-10 mm  11-20 mm (T2)  >20 mm (T3)  No Macroscopic Disease  N/A | 150  14  56  58  38 | 47.5  4.4  11.7  18.4  12 |
| FIGO Stage  IV  IIIC+IIIB+IIIA  IIIC  IIIB  IIIA  IIC  IIB  IIA  N/A | 53  248  230  14  4  8  4  2  1 | 16.8  78.4  72.8  4.4  1.3  2.5  1.3  0.6  0.3 |
| Grade  G3  G2  G1 | 281  28  7 | 88.9  8.9  2.2 |
| *BECN1* CNV  Gain  Amplification  Diploid  Shallow deletion | 23  1  50  242 | 7.3  0.3  15.8  76.6 |
| *BRCA1* CNV  Gain  Amplification  Diploid  Shallow deletion  Deep deletion | 23  1  51  240  1 | 7.3  0.3  16.1  76  0.3 |
| *TP53 status*  Mutated  Wild type | 303  13 | 96  4 |
| Primary Therapy Outcome  Complete Response  Partial Response  Progressive Disease  Stable Disease  N/A | 184  39  25  12  56 | 58.2  12.9  7.9  3.8  17.7 |
| Platinum *status*  Overall patients (316)  Sensitive  Late Resistant  Too-early Resistant  N/A  *TP53*-mutated patients (303)  Sensitive  Late Resistant  Too-early Resistant  N/A  *TP53-*wild type (13)  Sensitive  Late Resistant  Too-early Resistant  N/A | 128  62  31  95  125  57  31  90  3  5  -  5 | 40.5  19.6  9.8  30.1  41.25  18.81  10.23  -  23.06  38.47  -  38.47 |

**Supplementary Table S2. Gene mutations in TCGA ovarian samples with wild type *TP53*.** These genes appear mainly involved in cell receptor signaling, cell morphogenesis and cell adhesion and migration. Recurrent mutated genes are underlined and bold.

| Sample ID | No. of mutations | Recurrent mutated genes |
| --- | --- | --- |
| TCGA-09-2056-01 | 24 | **PALB2**, LZTR1, KDM5C, GAB2, LRP1B, EP400, ABCA3, CD5L, MS4A1, CDH9, FBN2, MEF2A, MID1, MYLK, OR1D2, OVGP1, PGK2, PI4KB, PSPH, **TTN**, COPB2, FADS2, CHST2, **MED13,** TRIOBP, CASC3, SLITRK3, **TRAPPC8**, WAPL, **NUP205**, SULF1, SYNE2, ZC3H7B, DMXL2, ZFPM2, RNF19A, UPF2, ISYNA1, RIF1, PCDHB2, VPS35L, **DSCAML1,** MEAK7, NLRC4, HPSE2, CSPP1, L3MBTL2, LRRIQ1, MT4, NT5C1A, ACCS, SCRT2, **NAV3,** NAF1, PKHD1L1, OPN4, OR51F2, OR10T2, FAM71A, PLB1, BTNL9, SYNE4, LRRN4, CCDC116, FYB2, PRR30, NXF2B |
| TCGA-10-0933-01 | 32 | RB1, RHOA, DTX1, MLLT3, CENPF, DNM1, FLG, MAOB, PSMB4, RCE1, SAE1, ATP9A, CAMTA2, MXRA5, CENPQ, DISP3, HHIP, VPS16, PEAK1, ZNF512, STRIP1, ZFAND2A, **MUC16**, ABCC12, CPXM2, KLHDC7A, CCM2L, ZFC3H1, AK9, PGBD2, OVCH1, FAM47FC |
| TCGA-13-0727-01 | 22 | TP53BP1, ACTL6A, ARSF, GRPR, NFRKB, PDE6B, BRD2, TAF2, ZNF41, ZNF99, SHANK2, ADGRL2, GAPVD1, ZBTB21, NTM, MIS18BP1, USP35, DOCK6, CDH24, KIF2B, SIAH3, UGT2A2 |
| TCGA-13-  1408-01 | 78 | BRCA1, EGFR, EPHA7, ERCC2, SALL4, AOC2, ARHGAP6, ADGRB2, CLCN4, PTK2B, EFEMP1, LRP1, MYBPC3, NKTR, PFAS, PHKA2, PTPRG, SCN4A, **SIM1**, SPG7, SPTB, SPTBN2, SYT5, **TAF12,** TIAM1, **TTN**, UBA1, IFRD2, PER2, CACNA2D2, RUBCN, TECPR2, ABCC5, LILRB3, RASA3, MAST1, PDZD2, GPATCH8, WSCD1, ZNF281, C2CD2, FBXO6, CPAMD8, SIGLEC9, NTMT1, F11R, PRR16, ARCHGEF38, WDR33, FRMD4A, DNAJC11, PCDHB15, PELI1, DLGAP3, NOM1, DNAI2, QSER1, TRPM3, ZMIZ2, HASPIN, MYO18B, FBF1, **NAV3**, ARAP1, AGAP3, EVC2, ASB11, BPIFB4, VPS13B, ANKRD23, **JAKMIP3**, TERIM59, C1QL4, PALM2-AKAP2, MAGEB17 |
| TCGA-13-  1477-01 | 43 | **PALB2**, LIFR, CLTCL1, RPTOR, CHRNB3, FCN2, GPM6B, IGHMBP2, KCNQ2, LRP2, MYL4, IK3C2A, PPP2R5D, RNASE3, ST5, **STK10**, THBS4, DOP1B, ZNF234, ENPP4, UNC13A, EDRF1, VPS54, NLGN3, TASOR2, VRTN, HJURP, PCDHB13, C11OPF16, **DSCAML1**, USP37, AGBL5, **TNN**, FNDC3B, GLB1L, ITM2C, TLN2, FGD3, NLRP4, DIPK2A, C6ORF136, ARHGAP30, RBMXL1 |
| TCGA-24-  1544-01 | 17 | F2, F13A1, MYH3, **SIM1**, TTC3, ZNF112, FZD7, KIF14, ARHGAP25, MVP, HDAC6, CCDC93, LRRC8C, NLRP12, PKD1L1, COL22A, MAP3K15 |
| TCGA-24-1565-01 | 24 | BRAF, **FBXW7**, **PRKDC,** CTR9, ANPEP, COL15A1, GEIA2, ISL1, KCND1, KRT12, MYH4, OR3A2, PITX1, SIGLEC1, STK4, PCDHA9, PLCB1, SLC2A2, EPB41L4B, TENM3, BEX1, LANCL2, ROBO3, PEAR1 |
| TCGA-24-2038-01 | 10 | **KRAS,** EMSY, MC2R, DNMBP, TMEM176A, THSD4, SSX2IP, OR4D2, WDR81, DNAH10 |
| TCGA-24-2293-01 | 53 | BRCA2, FOXF1, MYH9, NF2, PTCH1, STAT5A, SP140, KDM4C, BRIP1, ACLY, CACNA1E, CASP5, ACE, DPAGT1, CELSR2, EPS8, AFF2, GRB14, MYBPC2, **TTN**, ZNF91, DNAJA3, **THRAP3**, SRRM1, TCIRG1, WDR4, MTF2, MINAR1, CLEC16A, UBR4, ZNF285, KIF21A, PRR11, LMBRD1, PCDHGB7, PCDHGA7, FAM217B, **C19ORF57,** EPPK1, KBTBD7, KCNH7, **MUC16**, PXDNL, FREM1, ZXDB, BRWD3, OR4C12, SLC35B2, STUM, OR11L1, TMEM200B, CCDC88C, POTEE |
| TCGA-25-1316-01 | 14 | **KRAS, FBXW7,** ADCYAP1R1, CFB, CDH10, CDH17, IGHG2, KCNJ11, ABCB11, SLC39A6, TRIM39, **KBTBD7**, MMP21, OR4L1 |
| TCGA-25-1328-01 | 10 | CEACAM7, CLCNKB, DTNA, TBC1D25, DNAH11, WSCD2, TAS2R16, PCDH, ADGRV1, CEP19 |
| TCGA-25-2408-01 | 14 | CDKN1B, ERC1, CRYBG1, HES1, RAP1GAP, NDST2, ABCA9, PHF3, UACA, KCNH6, TEX26, OR14C36, PRR19, FAM45A |
| TCGA-61-2095-01 | 107 | **NRAS**, EIF1AX, FANCC, FURIN, SERPINB4, KAT6B, RNF213, CYP1A1, CYP2A6, DBH, DCTD, DOCK1, DPYSL3, EVPL, GP5, AGFG2, MUC4, MYO9B, SLC22A18, PFKFB4, PKNOX1, RNASE1, SCP2, **STK10**, TPBG, TRAF1, WIPF1, MAPKAPK3, RANBP3, DNAH17, UNC5C, CTSF, KSR1, CTDP1, ZMYM6, PIGB, IGDCC3, H6PD, SOX13, EPM2AIP1, TOM1, ARL4A, CNKSR1, TCIRG1, LAMC3, TACC3, TREH, ADNP2, **TRAPPC8,** PDZRN3, UHRF1BP1L, **NUP205**, SYNE1, ATP13A2, WBP1, KCNE4, NPAP1, TBC1D22A, CHD5, ANKRD2, FAM178B, MCM10, GPRC5D, CHRNA9, SLC7A10, CYP26B1, SH3GLB2, SDHAF3, ATP8B2, KLHL14, JCAD, DHX37, GRHL3, CCDC90B, UBE2O, **TNN,** RASL11B, **C19ORF57**, GAL3ST4, AKNA, KAZALD1, RILP, SOX7, QRICH2, **KBTBD7**, TCHP, SSH2, GAL3ST3, SIGLE12, GCNA, RP1L1, DNTTIP1, TBC1D16, NKAIN4, SGPP2, TRAM1L1, ATP8B3, TTC16, GLIS3, ADGRG5, PCSK9, **JAKMIP3,** CAVIN1, EPHA6, ZNF546, ZNF324B, FRG2. |

**Supplementary Table S3. Correlation between CNV of *BECN1* and *BRCA1*.**

| ***BECN1***  **CNV** | ***BRCA1* CNV** | | | | | |
| --- | --- | --- | --- | --- | --- | --- |
|  |  | Amplification | Gain | Diploid | Shallow (heterozygous) deletion | Deep (homozygous) deletion |
|  | Amplification | 0 | 0 | 1 | 0 | 0 |
|  | Gain | 0 | 23 | 0 | 0 | 0 |
|  | Diploid | 0 | 0 | 50 | 0 | 0 |
|  | Shallow deletion | 1 | 0 | 0 | 240 | 1 |
|  | Deep deletion | 0 | 0 | 0 | 0 | 0 |
|  | Total | 1 | 23 | 51 | 240 | 1 |

**Supplementary Table S4. Clinical outcome of patients bearing the ovarian tumor with wild type *TP53*.** Note: Compare to Wong *et al* 2013 (in which there were 15 *TP53* wild type patients), in the present study the cohort includes only 13 *TP53* wild type patients and 2 *TP53* mutated patients (TCGA-13-0755 and TCGA-25-2042).

|  | Sample ID | Overall survival *status* (months) | Disease-free *status*  (months) | Platinum *status*  (platinum-free interval in months) |
| --- | --- | --- | --- | --- |
| 1 | TCGA-24-2038-01 | Deceased (44.45) | Recurred/Progressed (38.41) | N/A |
| 2 | TCGA-25-1328-01 | Deceased (66) | Recurred/Progressed (8.02) | Resistant (1) |
| 3 | TCGA-13-0727-01 | Deceased (15.18) | Recurred/Progressed (8.31) | Resistant (4.3) |
| 4 | TCGA-24-2293-01 | Deceased (16.62) | N/A | N/A (refractory, 0) |
| 5 | TCGA-24-1544-01 | Deceased (26.94) | Recurred/Progressed (21.12) | Sensitive (14.5) |
| 6 | TCGA-24-1565-01 | Deceased (10.25) | Recurred/Progressed (4.76) | Resistant (N/A) |
| 7 | TCGA-09-2056-01 | Living (12.45) | Disease Free (12.45) | Sensitive (7.6) |
| 8 | TCGA-61-2095-01 | Deceased (61.6) | Recurred/Progressed (14.62) | N/A |
| 9 | TCGA-13-1477-01 | Deceased (54.57) | Recurred/Progressed (7.69) | Resistant (1) |
| 10 | TCGA-25-2408-01 | Deceased (30.95) | Recurred/Progressed (10.94) | N/A |
| 11 | TCGA-25-1316-01 | Deceased (42.02) | Recurred/Progressed (9.07) | Resistant (1.9) |
| 12 | TCGA-13-1408-01 | Living (5.91) | Recurred/Progressed (N/A) | N/A (refractory, 0) |
| 13 | TCGA-10-0933-01 | Deceased (14.65) | Recurred/Progressed (11.7) | Sensitive (7.3) |
